# High Affinity, Developability and Functional Size: The Holy Grail of Combinatorial Antibody Library Generation

**DOI:** 10.3390/molecules16053675

**Published:** 2011-05-03

**Authors:** Dirk Ponsel, Julia Neugebauer, Kathrin Ladetzki-Baehs, Kathrin Tissot

**Affiliations:** MorphoSys AG, Lena-Christ Strasse 48, 82152 Martinsried, Germany; Email: Ponsel@morphsys.com (D.P.); Ladetzki-Baehs@morphosys.com (K.L.-B.); Tissot@morphosys.com (K.T.)

**Keywords:** phage display, library

## Abstract

Since the initial description of phage display technology for the generation of human antibodies, a variety of selection methods has been developed. The most critical parameter for all *in vitro*-based approaches is the quality of the antibody library. Concurrent evolution of the libraries has allowed display and selection technologies to reveal their full potential. They come in different flavors, from naïve to fully synthetic and differ in terms of size, quality, method of preparation, framework and CDR composition. Early on, the focus has mainly been on affinities and thus on library size and diversity. Subsequently, the increased awareness of developability and cost of goods as important success factors has spurred efforts to generate libraries with improved biophysical properties and favorable production characteristics. More recently a major focus on reduction of unwanted side effects through reduced immunogenicity and improved overall biophysical behavior has led to a re-evaluation of library design.

## 1. Introduction

Until the invention of phage display, immunization and murine hybridoma technology were the only methods available for the generation of antibodies. Hybridoma technology however has its limitations when the antigens are toxic or non-immunogenic in mice. More importantly, however, generation of antibodies to self-antigens can be challenging, especially when there are high sequence homologies between the human and the respective murine antigen. *In vitro* antibody selection methods, such as phage display, have set a new standard. An important advantage of these antibody generation technologies is the absence of technical limitations, such as the choice of antigen and the ability to tailor both affinities and cross-reactivities to what is required. The past decades have seen a rapid development of large antibody repertoires and display methods, namely phage display, ribosomal display and bacterial, yeast or mammalian cell surface display. Today a multitude of antibody libraries exists that differ both in design and means of construction. The focus of this review will be on antibody libraries developed to isolate therapeutic antibodies. It is in the nature of the subject that commercial libraries play a major part.

One of the greatest challenges in library selection as compared to *in vivo* methods is the absence of somatic hypermutation (SHM) which enables nature to create high-affinity antibodies. Different affinity maturation strategies are available to overcome this limitation. All of these methods are based on introducing a certain degree of diversity into selected, moderate affinity candidates, followed by repeated selection with increased selective pressure. Affinities that can be achieved directly from a combinatorial library, without affinity maturation, are expected to correlate, to a certain degree, with the size of the library. The probability of identifying high-affinity antibodies thus increases with library size [please refer to 1]. Functional library size, however, matters more than absolute library size and this can vary dramatically depending on the design and means of construction. 

Another major challenge of *in vitro* selection methods is to deliver therapeutic antibodies that are developable and safe in human. Developability encompasses several different aspects of a therapeutic antibody: production, manufacturability, formulation interactions as well as the resulting shelf life. These parameters influence the costs of goods, but more importantly, affect dosing, modes of administration and can have an impact on drug safety. Protein properties responsible for developability include structural, colloidal and chemical stability, all of which are affected by the primary sequence and the state of potential posttranslational modifications. However, not all of these properties can be predicted from the primary sequence. For example, while apparent structural stability is often dominated by the composition of the framework regions, the aggregation propensity can be influenced strongly by CDR (complementary determining region) composition. 

Immunogenicity is another important aspect of a therapeutic antibody. There is one clear trend among monoclonal antibody candidates which have entered clinical phases within the last 30 years: to be as human as possible. In 1990 only 11.5% of all antibodies in clinical development were fully human, but this number has increased to 45% by 2000 [2]. This development is driven by the impact of immunogenicity on three aspects of therapeutic antibodies: pharmacokinetics, safety and efficacy. 

A major concern in the development of therapeutic antibodies is the generation of anti-drug antibodies (ADA) in patients following multiple dosing. ADAs may cause the following complications: drug-binding, which may significantly enhance antibody clearance, thus lowering half-life, and drug-neutralization, which is detrimental in a more direct way. 

ADA responses can result in a complete loss of efficacy in a significant percentage of the patient population. Since the ADA response is triggered in many cases by protein sequences that are recognized as foreign by the patients’ immune system, the aim is to avoid occurrence of such sequences as much as possible, even in the library design. Potential posttranslational protein modifications, such as glycosylation, deamidation, oxidation or isomerization of amino acid side chains constitute another factor that can influence antibody immunogenicity [3]. The simple credo has been “the more human, the less potential for immunogenic responses”. ADA-responses are, however, difficult to compare between different antibodies since they depend on the antibody sequence and format, but also on its formulation, route of application, dosing and finally on the target and patient population. A conclusion regarding the validity of the above-mentioned credo is thus difficult to draw with the limited amount of data available today. A recent review by Getts *et al*., discusses in depth this trend towards human antibodies with respect to immunogenicity and safety aspects [4]. 

In order to characterize a given antibody library, three main aspects have to be considered: the display method, the antibody fragment used as scaffold and the source of the sequence diversity. This review gives an introduction into the field with a focus on antibody-based phage display libraries for the generation of therapeutic antibodies, many of them developed in an industrial setting. To put everything into context, it will briefly describe the display methods used most frequently and touch on different antibody formats currently in use for display and selection.

## 2. Display Methods for Combinatorial Libraries

**Figure 1:**
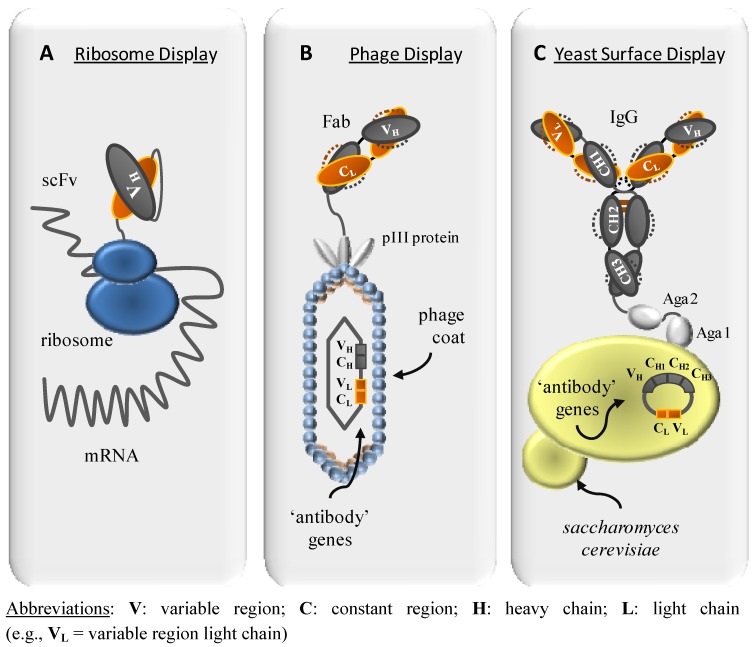
Display methods as tools for antibody selection. Antibody (e.g., scFv, Fab, IgG) display methods include ribosome and phage display as well as cell surface display, such as yeast display. A: Ribosome display; *in vitro* evolution and selection method for scFv fragments. B: Phage display; protein selection method, where antibody derived fragments are genetically fused to pIII phage-coat protein and presented on the surface of filamentous phage. C: Yeast display; cell surface selection method, capable of presenting full length IgGs. The a-agglutinin yeast adhesion receptor, composed of two proteins Aga1 and Aga2, is used to display recombinant proteins on the surface of *S. cerevisiae*.

A variety of display and protein interaction systems have been evaluated as selection methods for antibody-antigen interaction. Commonly used methods for the generation of therapeutic antibodies from combinatorial libraries all comprise a display of the antibody, either on the surface of phages, of eukaryotic cells such as yeast, or on ribosomes following *in vitro* transcription (Figure 1). All display methods have two major advantages in common: (1) they can be applied to a wide selection of antigens and (2) the conditions and thus the selective pressure can be tailored to the requirements. In contrast, intracellular selection methods, such as yeast-two-hybrid system or protein complementation assay depend on intracellular expression of the target. The control over selective pressure is thus limited by the given biological system [5,6,7]. Those latter selection systems will not be addressed further.

### 2.1. Ribosome Display

The concept of ribosome display was initially proposed by Mattheakis and coworkers and established by Hanes and Plückthun as a selection method for antibody scFv fragments [8,9]. 

In contrast to display methods employing phage display or surface expression on cells, ribosome display is an evolution and selection system that takes place completely *in vitro*. Due to relative instability of the RNA and the ribosomal complex, the method is technically more challenging than e.g., the very robust phage display technology. Concerning the antibody format, it is limited to single-chain proteins, usually the scFv. Since no transformation is necessary, very large libraries (10^12^) have become accessible in a single step. Several cycles of selection and mutagenesis/recombination are possible in a short time, because the PCR (polymerase chain reaction) amplification can easily be coupled with random mutagenesis or PCR-mediated recombination. An additional advantage of ribosome display compared to conventional phage display is the possibility to elute mRNA from antigen-bound ribosomal complexes by the addition of EDTA. This mild elution condition results in the dissociation of the ribosomal complex and thus ensures complete recovery even of very high affinity antibodies, which often can be difficult to achieve with elution strategies based on disruption of antibody-antigen interaction. 

### 2.2. Yeast Surface Display

Yeast cell surface display of scFv antibodies was first established by Boder and Wittrup in 1997 and its applicability for the selection from large libraries was shown by Feldhaus and coworkers [10,11,12]. In contrast to phage display, full length IgGs can successfully be displayed on both yeast cells [13 (poster presentation)] and mammalian cells [14]. The advantages of using full length IgG during selection are two-fold: First of all, selection takes place in the envisioned therapeutic format. Second, potential impairment of affinity or potency, due to antibody format changes is unlikely to occur. In addition, cell surface display is compatible with FACS-sorting techniques, which allows antibody selection close to ‘natural’ conditions in solution and parallel assessment of multiple parameters like antibody expression levels, number of bound antigen, or cross-reactivities.

A limitation of yeast and similarly all other eukaryotic cell surface systems is the limited transformation efficiency of those cells, which sets upper limits on the library size that can be realized. The first yeast display libraries generated were thus of moderate size, in the range of 10^5^–10^7^ [10,15]. Yeast mating, a feature of the haploid/diploid lifecycle of yeast allows the generation of larger libraries (Fab or IgG) in yeast from two separate vectors and is also amenable to chain shuffling for affinity improvement [12].

### 2.3. Phage display

Smith established the heterologous expression of peptides on the surface of filamentous phages [16]. Since then, phage display has become a widely used method for the display of both peptide and protein libraries. Antibody formats that can be displayed on phages are limited by their ability to express efficiently in the periplasm of *E. coli*. Two types of phage systems can be distinguished: phage vector and phagemid vector based display systems. The phage vector system consists of the entire phage genome with the antibody gene inserted as a fusion to a phage surface protein (in most cases gene III). The phagemid system consists of two components: a phagemid carrying the phage surface protein-antibody fusion and a so called helper phage. 

A major advantage of phagemid vectors is their smaller size and ease of cloning, allowing for large library sizes. The main difference between both systems lies in the display level of the antibodies. 

While every phage produced by a phage vector system carries 3–5 copies of the antibody on its surface (multivalent display), the display on phagemid systems is monovalent with <10% of the phages carrying an antibody on the surface [17]. Sometimes both systems are used sequentially, taking advantage of the avidity effect of multivalent display for the first selection round combined with higher stringency of monovalent display in later selection rounds. To improve antibody display levels in the phagemid system, several groups have generated helper phage carrying different mutations affecting gene III [18,19,20,21,22,23,24]. The hyperphage e.g. carries a gene III deletion which leads to multivalent display in combination with a phagemid [21], allowing an easy switch from multivalent to monovalent display within one system.

Since the initial application of phages for the display of antibody fragments by Winter as well as Burton and Lerner a large variety of different phage antibody libraries has been constructed [25,26]. Similar to cell surface display systems, phage library sizes are limited by the transformation efficiency of the bacterial host. In order to circumvent this limitation many repeated electroporations are necessary to create large repertoires [27]. 

With both advantages, such as robustness, as well as certain limitations, phage display has become one of the most frequently used display methods for combinatorial antibody libraries [28]. Due to high stability of the phage, phage display technology is amenable to an extensive range of conditions that can be adapted to drive the selective pressure in the desired direction. An extreme example is the use of high temperature or concentrations of denaturant to select for high stability of the displayed protein [29].

## 3. Antibody-derived Fragments Used in Phage Display

The antibody formats used for phage display initially by the groups of Winter and Lerner were single-chain Fv (scFv) and Fab-fragments [25,26,30]. Both formats are used due to their favorable expression properties in the periplasm of *E. coli* and their ability to display on phages, which cannot be achieved with full length IgG. 

ScFv-fragments are only moderately stable on average and often have a high tendency to form multimers as well as aggregates. Fab fragments in contrast are stabilized significantly by an additional interface of the constant domains [31]. They have been found to possess comparably higher structural stability, resulting in overall reduced aggregation and therefore a higher monomeric proportion. For these reasons, Fab fragments have replaced the scFv as display format in many of the more recent phage libraries. Besides the above-mentioned advantages which are important factors during selection and screening processes, the Fab format allows conversion into full length IgGs without impairment of function.

While scFv and Fab are the most commonly used antibody formats in phage display today, additional antibody derivatives have come into play that are based on engineered single antibody domains. The concept of single domain antibodies (sdAb) derived from antibody variable domains has been introduced over 20 years ago by Ward and coworkers [32]. 

Like many scFv fragments, sdAbs are however unstable, with a tendency to aggregate and it took several more years until the technology of domain antibodies became of therapeutic and commercial relevance. 

The antigen binding site of antibodies from camelids and also cartilaginous fish frequently comprises the heavy chain variable domain only (referred to as VHH to distinguish it from classic VH). In contrast to isolated human VH domains, VHH domains are well expressed from bacteria and yeast and show high resistance towards aggregation, even at elevated temperatures [33,34]. The presence of an enlarged CDR1 and CDR3 in VHHs and loops that exhibit alternative canonical structures increases the structural repertoire of the antigen-binding site and compensates for the absence of the three VL CDRs. 

The experience gathered with naturally occurring sdAb libraries from immunized camels and llamas, together with the results of structural studies on camelid and camelized domains and the characterization of their thermodynamic properties was the basis for the rational design of camelized domain antibodies [33,35,36,37,38,39,40,41,42]. Like the camelid VHH, they proved to be a small, robust and efficient single domain binding unit. In analogy to the structures observed for camel VHHs, the changes reduce hydrophobicity and thus prevent non-specific binding of camelized VH domains [38]. Concerning their use as antibody scaffold for phage display, the engineered domain antibodies fulfill the necessary requirements of high soluble expression in the *E. coli* periplasm.

The development of domain antibodies based on naturally occurring VHHs or variable heavy domains engineered in analogy, was motivated by three assumed advantages of small antibody fragments compared to full length IgGs: (1) More rapid and efficient tissue penetration (2) high stability and good expression in bacterial and yeast systems and (3) targeting of novel epitopes. 

The last point is based on the unusual structural characteristics of domain antibodies. The long CDR3 is at the basis of the domain antibodies´ unique ability to recognize epitopes that are normally out of reach for conventional antibodies. This was shown by the isolation of VHHs that bind into cavities of enzymes, such as lysozyme [43,44,45,46]. The crystal structure of an anti-lysozyme VHH showed that part of the long CDR3 loop of 24 amino acids inserts into the active site of the enzyme [47]. It was the first example of an antigen-binding site with a large protruding loop. On the downside, the small size and the missing Fc part enabling FcRn binding limits serum half-life *in vivo*. To circumvent this limitation, additional modifications such as PEGylation to increase molecule size and thus prevent kidney clearance are often required [48,49]. The increased size, however, may reduce tissue penetration and the costly modifications can diminish the competitive advantage gained by cheap bacterial expression. In addition to that, small antibody formats lacking the constant domains are devoid of effector functions. Their use is therefore limited to applications that do not require Fc-effector functions.

With the aim of overcoming certain limitations of the immunoglobulin fold as such, and to further explore the suggested advantages of antibody fragments mentioned above, a number of novel protein scaffolds have been used for library construction. Those novel scaffolds are, however, outside the scope of this review and will not be addressed further.

## 4. Design of Combinatorial Antibody Libraries

**Figure 2:**
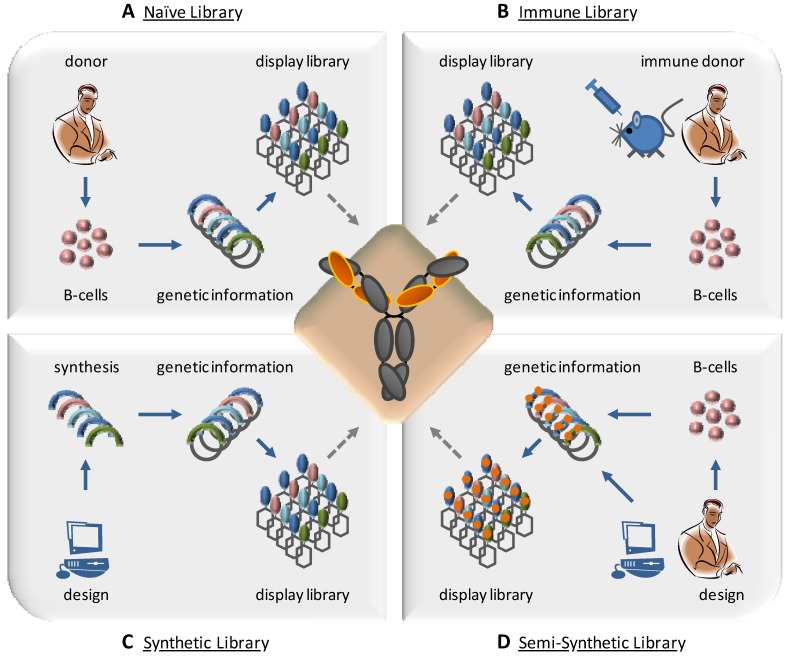
Four types of antibody libraries can be distinguished by source and design. A: Naïve libraries are amplified from a natural source, such as primary B-cells of non immunized donors. One library can be used for a wide variety of antigens. B: Immune libraries are generated from B-cell derived antibody repertoire of immunized or immune donors. Libraries are predisposed for a limited panel of antigens.C: Synthetic libraries are based on computational *in silico* design and gene synthesis. CDR design and composition is precisely defined and controlled.D: Semi-synthetic libraries comprise both CDRs from natural sources as well as in silico design of defined parts.

Four types of antibody libraries can be distinguished with respect to the source of library sequences: Immune, naïve, semi-synthetic and synthetic libraries (Figure 2). While the first two are based entirely on naturally occurring sequence diversity, synthetic libraries are diversified according to design. Semi-synthetic libraries combine natural diversity for certain aspects of the library with *in silico* design. Although the combinatorial aspect is most prominent in semi-synthetic and synthetic libraries, naïve and immune libraries also feature certain combinatorial aspects, e.g. the random pairing of heavy and light chains. For representative examples of different phage display libraries please refer to Table 1. 

Generation of large functional libraries is limited by transformation/transfection efficiency and the occurrence of frame shift mutants leading to non functional proteins. The cloning of large antibody libraries is therefore technically challenging, laborious and time consuming. 

The principle of *in vivo* recombination of the light and heavy chain gene of an antibody was first described by the group of Greg Winter as a means to increase library size [65]. A *Cre*/*loxP* two vector system was used, with a donor plasmid vector encoding the heavy chain and an acceptor fd phage vector encoding the light chain. Both heavy as well as light chain gene are flanked by *loxP* sites leading to a site-specific recombination *in vivo*. By-passing the limitation of transformation efficiency with infection, this system allows the generation of very large libraries. Griffiths and coworkers used this system to generate a phage display library of 6.5 × 10^10^. Sblattero and Bradbury used a modified version where both the heavy and light chain V-genes were located on the same phagemid separated by a *loxP* site. Starting out with a relatively small initial library with a size of 7 × 10^7^ they were able to generate a library of 3 × 10^11^ *in vivo* by site-specific recombination [66].

**Table 1:** Representative examples of phage display antibody libraries (scFv and Fab format) published. All phage display libraries are summarized in brief by key parameters as framework and CDR ‘source’, library size and highest affinities described. Selected references from the literature are given for each library.

Library Name	Format	Framework	CDR Source	Library Size	Published Affinities	Reference
-	scFv	immune	immune		nM	[50]
RAB-03/04-G01Crucell		immune	immune	1 × 10^7^		[51]
-		naïve	naïve	>10^7^	low pM	[26,52]
CAT1.0 MedImmune		naïve	naïve	1.4 × 10^10^	low to sub-nM	[27]
CAT2.0 MedImmune		naïve	naïve	1.3 × 10^11^		[53]
-		• 49 VH frameworks • single VL framework	• synthetic CDR-H3	1 × 10^7^	µM	[54]
-		• 49 VH frameworks • single VL framework	• synthetic CDR-H3	10^8^		[55]
-		• 49 VH frameworks • 7 VL frameworks	• synthetic CDR-H3	3.6 × 10^8^	100nM to µM	[56]
-		• single VH framework • single VL framework	• synthetic CDR-H3 and CDR-L3	>3 × 10^8^	nM	[57]
n-CoDeR® Bioinvent	scFv /Fab	• single VH framework • single VL framework	• natural	10^9^-10^10^	sub-nM	[58,59]
Genentech	Fab	• single VH framework • single VL framework	• synthetic CDR-H1 -H2, -H3, (-L3)	up to 10^10^	low nM	[60,61]
-		• 49 VH frameworks • 26 VL_k frameworks • 21 VL_l frameworks	• synthetic CDR-H3	6.5 × 10^10^	up to single digit nM	[62]
Dyax		• single VH framework • natural VL	• natural CDR-H3 • synthetic CDR-H1/-H2	1 × 10^10^	sub-nM	[63]
HuCAL GOLD®MorphoSys		• synthetic (consensus sequence)	synthetic	1.6 × 10^10^	pM	[64]
HuCAL PLATINUM®MorphoSys		• synthetic (close to germline)	synthetic	4.5 × 10^10^		

Another important aspect is functional library size, which is limited by the occurrence of deletion mutants caused by frameshifts or stop codons introduced during diversification. Tremendous progress has been made by construction of vector systems comprising a positive selection for protein integrity. This work was pioneered by Little *et al*. who used the *bla* gene (β-lactamase) as a selection tool for in frame, full size protein expression. In this system, a scAb-*bla*-fusion protein was introduced into a phagemid. Following protein expression, only library clones comprising functional scAb-β-lactamase gene products not affected by unintended frameshifts or internal stop codons survived the respective antibiotic selection [67].

### 4.1. Immune Libraries

Immune libraries represent a special case, since they are derived from immune donors and are thus predisposed for recognition of certain antigens. Due to this predisposition, they are usually comparatively small in size. In contrast to naïve libraries, however, immune libraries are not well suited for the identification of antibody fragments against a large panel of antigens, especially self-antigens. 

The B-cell repertoire of mice immunized with the keyhole limpet hemocyanin (KLH) coupled *p*-nitrophenyl phosphonamidate (NPN) antigen was the source of the first immune library [50]. Rearranged immunoglobulin genes of IgG1 heavy and kappa light chains were amplified *via* PCR using sets of degenerate primers. The resulting combination of heavy and light chains was random and did not reflect the original pairing. After screening of the scFv phage display library against the NPN-antigen about 100 specific clones were identified with affinities in the nanomolar range. Since then, immune libraries have been generated from a variety of different species [68,69]. 

For human immune libraries, two main applications are the selection of virus neutralizing antibodies from infected patients and the identification of tumor specific antibodies from libraries of cancer patients [25,51,70,71,72]. As an example, Kramer and coworkers described the generation of immune libraries for the selection of antibodies specific for the rabies virus glycoprotein. Libraries were constructed based on four different donors immunized with rabies vaccine. The libraries were of moderate size, in the order of 1 × 10^7^. 147 specific clones, and among those 39 virus neutralizing scFvs could be identified. Although these libraries were constructed based on random pairing of single VH and VL chains, a bias was noted towards certain VH/VL pairings. Additionally, a preferential use of certain germlines (e.g., VH3_30) and very similar CDR-H3 sequences was found [51]. 

Up to 75% of the immunoglobulins found in the sera of camelids, such as camels, dromedaries or llamas are functional antibodies consisting solely of two paired heavy chains containing a VHH domain each [73]. Muyldermans and coworkers at Ablynx make use of those naturally occurring camelid VHH antibodies for the generation of Nanobodies^®^ [74,75]. A Nanobody^®^ differs from a human heavy chain variable domain in about ten amino acids, four Nanobody^®^-specific amino acids in the framework-2 region and a longer third antigen-binding loop (H3) folding over this area. Nanobodies^®^ in the nanomolar range are routinely isolated from immunized animals against a wide range of targets. Thus, the affinities of the selected VHH domains for their respective antigens are in a similar range as the affinities of monovalent scFv of Fab fragments derived from comparable libraries [76].

### 4.2. Naïve Libraries

Naïve libraries are derived from primary B-cells of non-immunized donors. Naturally rearranged variable region genes have been used to construct large repertoires of up to 10^11^ members. In contrast to immune libraries, one single library can be used for the generation of antibodies against all types of antigens, including toxins and self-antigens. With smaller libraries, affinities are typically in the micro- to lower nanomolar range. Large libraries have delivered affinities down to the sub-nanomolar range. James Marks and others in the lab of Greg Winter pioneered the work with naïve antibody libraries. In 1991 they published the construction of diverse libraries of immunoglobulin heavy and light chain variable genes from peripheral blood lymphocytes (PBLs) of non-immunized donors by polymerase chain reaction (PCR) amplification [26]. 

The combinatorial library (> 10^7^ members) was made by randomly combining heavy and light chain V-genes using PCR. Sheets and coworkers combined the light chain V-gene repertoire generated by Marks and coworkers with a newly generated heavy chain V-gene repertoire to a large naïve library with a library size of 6.7 × 10^9^ [26]. B-cells from the spleens of three different donors as well as from PBLs of two different donors were used as source for the heavy chain V-gene repertoire. This heavy chain V-gene repertoire was cloned into a vector *via* RT-PCR and then combined with the already existing light chain V-gene repertoire for the scFv library *via* overlap extension PCR [77]. Both libraries were used for selections against different protein antigens and haptens. Whereas the smaller library resulted in scFvs with affinities in the low micromolar range, affinities as high as subnanomolar were reached using the larger library created by Sheets and coworkers [26,77]. The identified V-genes were in part nearly identical to known germline genes, while others were more heavily mutated [26]. 

The CAT1.0 library (Cambridge Antibody Technology, now part of MedImmune/AstraZeneca) is the next big step in further development of that technology and the first large naïve antibody phage display library [27]. Rearranged antibody V genes for heavy and light chain genes were amplified from B-cells of 43 human donors and randomly combined into scFvs resulting in a library with a size of 1.4 × 10^10^. 

The library was used for selections on a panel of different antigens and yielded 3-20 different scFvs per antigen. Following selection, again the VH3 framework was overrepresented, followed by VH1 and VH4 frameworks. 

The CAT2.0 library was a further extension of CAT1.0 [53]. Additional new sub-libraries had been generated, to increase the total library size to 1.29 × 10^11^. The library was successfully used to identify antibodies against 28 different antigens (27 human and one viral antigen), with a mean of 119 unique scFvs per antigen. The potency (EC_50_ or IC_50_) of the lead scFvs *in vitro* ranged from 0.09–250 nM. Analysis of the framework usage and diversity of the library showed that almost the entire natural VH repertoire was represented in the CAT2.0 library (48/49 VH, 28/30 V_lambda, 31/35 V_kappa gene segments). During selection particular gene segments like VH1 as well as lambda light chains were enriched over others. The VH3 framework was used frequently but there was no further enrichment during selection. While there was no bias before selection towards a particular VH-VL pairing, after selection a preference for certain VH-VL pairings was observed, the most abundant pairing being VH1-Vlambda1. Other groups used similar approaches to generate large naïve libraries and also succeeded in isolating antibody fragments with affinities down to the picomolar range [77,78].

### 4.3. Synthetic and Semi-Synthetic Libraries

The fundamental difference between immune or naïve libraries and synthetic libraries consists in the origin of the sequences used to build the library. While immune and naïve libraries are amplified from a natural source, synthetic library parts are diversified by design. In synthetic libraries the antibody diversity is designed *in silico* and then synthesized in a controlled fashion. 

The ratio of naturally-derived and synthetically-designed parts varies in different libraries from semi- to fully synthetic. An advantage of synthetic diversification is that the composition of the CDRs can be exactly defined and controlled. 

Synthetic libraries can further be grouped according to the acceptor frameworks used, the origin or design of the sequence diversity within the CDR regions and the method used for building the library. 

The first semi-synthetic libraries used a variety of different framework genes to keep diversity high [55,56,62,79]. In 1992 Hoogenboom and Winter described semi-synthetic scFv-antibody phage display libraries, comprising 49 germline VH sequences and a single V_lambda 3 light chain sequence [79]. Five or eight residues in the CDR-H3 were randomized in a PCR-based approach to generate libraries with a size of 1 × 10^7^. Subsequent libraries were based on the same set of acceptor VH frameworks, with the addition of 26 kappa and 21 lambda sequences [62]. Length variability was introduced in the CDR3s and diversity generated through randomization of 4-12 residues in CDR-H3, 1-3 residues in kappa CDR-L3 and 0-5 residues in lambda CDR-L3. In parallel, library size increased over three orders of magnitude up to 6.5 × 10^10^. 

In a similar approach, de Kruif and collaborators used all 49 human germline VH genes and seven different light chain genes (4 V_kappa, 3 V_lambda) with CDR-H3 length variability between 6-15 residues to construct a library of the size of 3.6 × 10^8^. 

The shorter CDR-H3s of six amino acid length were fully randomized, whereas for the longer CDR-H3s the design included a stretch of fully randomized amino acid residues flanked by regions of lower diversity resembling human antibody sequences [56].

While specific antibody fragments to both haptens and protein antigens could be selected from the smallest libraries, affinities were very moderate [79]. A strong bias towards the VH3 framework was observed. Using a ten times larger library, scFvs against 18 different antigens were selected [55]. All VH families except VH2 were found, but VH3 was strongly overrepresented. The large semi-synthetic library constructed by Griffiths and coworkers yielded even higher hit-rates of unique, specific clones after selection [62]. The resulting antibodies represented 17/49 VH fragments, 10/26 V_kappa segments and 9/21 V_lambda segments, including all of the major families. Fifty two different heavy/light chain pairings were identified, with several VH genes being promiscuous. Affinities of purified Fabs ranged from 3.8 to 217 nM (antigens: NIP-CAP, fluorescein, HGF/SF, NML1, NML9), showing that high affinities can be achieved with a large library. 

Accumulating results from selections done with the early libraries confirm the expectation that larger libraries yield more specific antibodies with higher average affinities. A second observation is that certain frameworks (predominantly VH1 and VH3) are over represented after selection by phage display, often beyond the ratio that can be expected from the input libraries. 

This observation, together with the desire to increase functional library size led to the development of single acceptor framework libraries. VH and VL frameworks were chosen based on the frequency of use and for their favorable stability and/or expression properties [57,63]. 

Libraries that are based on a single framework combination almost exclusively use the heavy chain framework VH3_23, a framework that is frequently found in human antibodies, pairs with almost all light chains, shows a good expression in bacteria and displays well on phages. 

### 4.4. Defined Frameworks: Maximal Diversity

Pini and coworkers used VH3_23 heavy chain and the light chain corresponding to the Vκ3_20 germline for its expression and stability properties [57]. Defined positions were randomized in CDR-L3 and CDR-H3, resulting in a library size of >3 × 10^8^. 88% of the clones were shown to express a functional antibody and scFvs were selected with monovalent affinities down to 10 nM [57]. 

**Figure 3:**
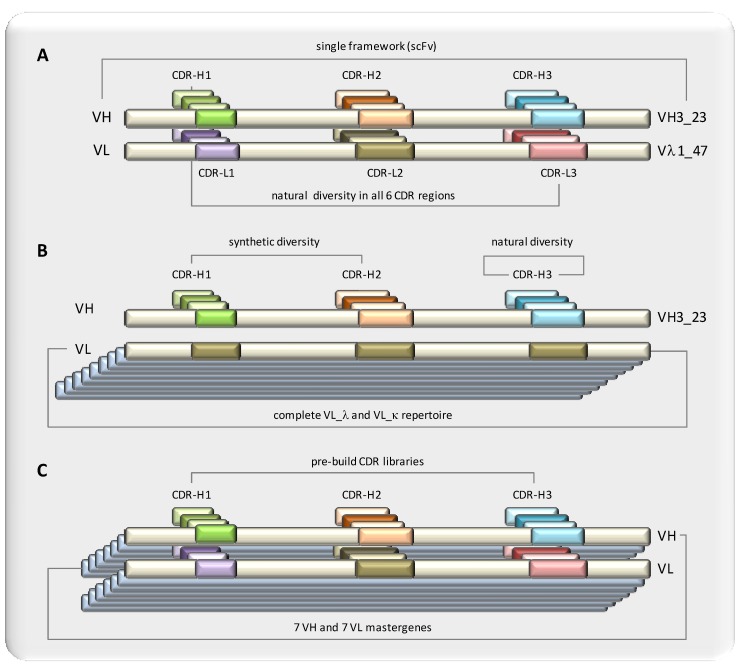
Representative examples of semi-synthetic and fully synthetic library design. Semi-synthetic libraries combine synthetic design and natural sources to different degrees whereas fully synthetic libraries are based on in silico design and de novo synthesis. Three examples are schematically shown.Library A comprises natural diversity within all CDR regions integrated into one selected single scaffold. CDR sequences are gained *via* PCR amplification from all naturally occurring frameworks (adapted from semi-synthetic N-CoDeR^® ^library).Library B comprises synthetic diversity in CDR-H1 and –H2 combined by natural diversity within CDR-H3. Whereas one single heavy chain framework was selected, the whole light chain repertoire has been used (adapted from semi-synthetic Dyax library). C: The fully synthetic HuCAL^®^ library concept is based on consensus sequences representing major germline families yielding 7 VH and 7 VL mastergenes. CDRs are designed to match amino acid composition of naturally occurring antibodies.

The principle of the n-CoDeR^®^ scFv library (BioInvent) is also based on the use of a single, stable framework, namely VH3_23, combined with V_lambda (DPL3) (Figure 3) [58,59]. In contrast to the library by Pini, natural CDRs were used for the generation of diversity. Sequences encoding *in vivo*-formed CDRs from rearranged immunoglobulin genes of different germline origin were combined into one single master framework by amplification of CDRs with primers and overlap extension. Due to the fact that only one fixed framework was used, CDRs from other germlines were combined with this fixed framework. CDRs were amplified from B-cells of several donors and from several different tissues including lymph nodes and PBMCs. The assumption was that these CDRs might contain fewer T-cell epitopes compared to an *in silico* design, due to the proofreading mechanism they underwent *in vivo*. The initial library had a size of 2 × 10^9^, which has increased by one order of magnitude and delivered affinities in the sub-nanomolar range. 

A similar framework was also used by Lee and coworkers to construct libraries with completely synthetic CDRs displayed on a single scaffold [60]. They based their library on the framework of the humanized 4D5 antibody (Herceptin^®^), a framework derived from VH3_23 and V_kappa). A design strategy of mimicking natural diversity using tailored codons was used to closely mimic the human repertoire. Different library generations were described and it could be shown that the restricted diversity in CDR-H1 and CDR-H2 improved the performance of the library whereas highly variable CDR-H3 sequences were advantageous. Affinities in the low nanomolar range were achieved using these libraries.

Hoet and coworkers at Dyax combined synthetic diversity in CDR-H1 and -H2 with length and sequence diversity in CDR-H3 from natural origin (Figure 3) [63]. CDR-H1 and -H2 repertoires were designed based on analysis of germlines genes with introduction of hot spot mutations. Those variable parts were constructed with codon-based oligonuceotides to avoid unwanted amino acids and stop codons, with the aim to increase the functional library size. VH3_23 was used as heavy chain acceptor and combined with the full lambda and kappa light chain repertoire. The natural diversity of the CDR-H3 and light chains originated from B-cells of 35 donors having various autoimmune diseases and 10 healthy donors in order to maximize the diversity. The light chain and all three heavy chain CDRs are independently replaceable modules to facilitate affinity maturation. From the phagemid Fab library with a size of 3.5 × 10^10^ and a phage library with a size of 1.0 × 10^10^, affinities in the subnanomolar range were obtained. 

In a special application of the same principle, Schoonbroodt and coworkers later generated a library focusing on anti-carbohydrate antibodies [80]. The goal of this library was to generate a library tailored for the generation of antibodies recognizing negatively charged carbohydrates by introducing basic residues at defined positions. The design of the CDR-H3 was based on sequence alignments of known carbohydrate specific antibodies, while the CDR-H1 and CDR-H2 as well as the VL were unaltered compared to the original library. The library was successfully tested on two human charged carbohydrate targets, heperan sulfate and 6-sulfosialyl Lewis X core. 

The HuCAL^®^ (Human Combinatorial Antibody Library) libraries (MorphoSys) have taken the synthetic library concept one step further [64,81,82]. Library design is based on consensus sequences representing major germline families rather than using single germlines (Figure 3). The principle of consensus sequences was based on the finding that canonical sequence approximation is successful in predicting stabilizing mutations in the immunoglobulin fold [83]. Since the different frameworks contribute to the structural diversity of human antibodies, this is taken into consideration by including a variety of frameworks, yielding a total of seven heavy chain and seven light chain frameworks, resulting in 49 potential combinations. 

Starting with HuCAL^®^ libraries comprising CDR3 diversification only, library sizes were increased by two orders of magnitude up to 1.6 × 10^10^ for the HuCAL GOLD^® ^version, with full diversification of all six CDRs. HuCAL GOLD^®^ delivered antibodies with affinities in the picomolar range, whereas best affinities were obtained in the single digit nanomolar range for the initial HuCAL^®^ library [64,81,82,84]. HuCALs’ synthetic CDR regions are designed to reflect the natural amino acid distribution and length variation at each position for each respective framework. To maximize functional library size CDR regions are synthesized *via* TRIM technology and a selection for the integrity of the antibody-construct is implemented using the above-mentioned β-lactamase selection system [67]. TRIM technology uses prebuilt trinucleotides instead of single nucleotides and enables a better control of CDR design and avoidance of unwanted amino acids and stop codons [85]. 

In the latest version, HuCAL PLATINUM^®^, framework sequences are as close as possible to the nearest germline sequence, excluding non-germline-positions caused by formation of consensus sequences. In parallel, functional sequence space in HuCAL PLATINUM^®^ was increased by omitting unproductive frameworks, such as VH4 and kappa4 and by addition of a supplemental VH3 framework. Re-evaluation of an extended set of sequence data available led to a new design of the CDR-H3 that replicates natural amino acid composition and length variability within CDRs more closely. While the CDR design of the HuCAL GOLD^®^ library was based on analysis of 2,460 sequence entries [64], sequence information increases >5-fold to 14,800 entries for HuCAL PLATINUM^®^. In addition to optimized mastergenes and new CDR-H3 design HuCAL PLATINUM^®^ considers a length dependent amino acid composition within the CDR-H3 region. 

Concomitantly, the occurrence of N-linked glycosylation sites in the CDRs has significantly been reduced to avoid this post-translational modification as a potential source of heterogeneity with implications on stability, function and immunogenicity.

### 4.5. Elucidating Minimal Requirements: Restricted Diversity

In marked contrast to the approach of maximizing diversity within the frame of the naturally occurring sequence space, a series of libraries was constructed utilizing only a subset of amino acids for diversification. Based on findings that naturally occurring, antibodies are often biased in favor of tyrosine and serine residues in their antigen binding sites Genentech developed antibody libraries with very limited diversity in the CDR regions. Solvent accessible CDR positions were randomized using only a subset of 12 [86], four [87] or only two [88] amino acids. From these libraries, with a diversification as low as 4 amino acid, specific Fab antibodies with single digit nano molar affinities have been selected for different antigens (e.g., VEGF) [87]. Crystallographic analyses as well as shotgun scanning confirmed the importance of the tyrosine residues for antigen binding [89].

### 4.6. Diversity Limited to Natural Design: Adimab Technology

The antibody library generated by Adimab for yeast cell surface display of full IgGs is a further interesting example in this trend of limiting and “humanizing” the randomization strategies used for library design. The antibody frameworks used to build the library represent a selection of fully human germline sequences that are most frequently used in humans. 

The design of the CDR regions is based on the analysis of the available sequence information and intended to reflect the human pre-immune diversity. It is a germline-based approach meant to preserve the integrity of actual human sequences by tailoring the diversification to each specific germline. The CDR-H3 is designed to account for the recombination of immunoglobulin gene segments (*i.e.*, V, D, and J) with the hope to reflect natural human pre-immune antibody diversity more closely than by a design based on calculated amino acid frequencies. The limitation of theoretical diversity compared to full randomization according to amino acid frequencies is considered to be an advantage, especially in the light of the limitation on library size inherent to yeast display technology. 

### 4.7. Camelized Human VH Domains: Synthetic Libraries

In parallel to the development of semi-synthetic and synthetic libraries based on scFv or Fab fragments, synthetic single domain antibodies libraries were established. Repertoires of camelized VH domains were created by randomization of residues within the CDR3 loop, with simultaneous variation in length. Tanha and coworkers constructed a human VH library based on a camelized VH sequence through complete randomization of 19 of the 23 CDR3 residues. From a library size of 2–6 × 10^7^, domain antibodies with affinities in the micromolar range were isolated [90]. From a repertoire of 2 × 10^8^ clones, Davies and coworkers selected camelized VH domains specific for hapten, peptide and protein antigens with affinities in the high nanomolar range [37]. 

In contrast to those camelized VH domains, the scaffold used by Reiter and coworkers for library construction was a native sequence of a monoclonal antibody with a unique VH/VL interface. The library, consisting of 4 × 10^8^ independent clones, was generated by randomization of nine amino acid residues in the CDR3 and yielded affinities in the nanomolar range [91]. 

As for the VHH domain libraries, the affinities published with these synthetic single domain libraries are in similar range as the affinities of monovalent scFv of Fab fragments derived from comparable libraries.

Domantis (GSK) further advanced the technology by developing a series of large and highly functional libraries of human VH and VL dAbs™, based on human germline sequences that were adapted to generate single domain antibodies (VH and VK). The domain scaffolds were selected for high stability and engineered for solubility and resistance to aggregation. Additionally, the generation process includes a stringent selection step (heat stress) to maintain the desired favorable properties: heating of VH domain-displaying phages leads to protein unfolding and potential aggregation. After cooling, intact VH’s are enriched by selection on Protein A, thus enriching for domain antibodies with reversible unfolding properties [92,93].

## 5. Maturation Strategies

*In vivo*, high-affinity antibodies are generated by the immune system through a combination of steps introducing diversity (somatic hypermutation) and a subsequent selection (clonal expansion). During antigen stimulated B-cell proliferation, the immunoglobulin locus undergoes a very high rate of somatic mutation. 

There are several different approaches to mimic these events *in vitro* to improve the affinity of antibodies obtained from combinatorial libraries. For *in vitro* affinity maturation, selected molecules are randomized to introduce diversity followed by selection with increased selective pressure to identify improved variants [94]. In general one can differentiate between targeted and non-targeted diversification strategies. 

### 5.1. Non-Targeted Diversification

Error prone PCR and the use of mutator *E. coli* strains are two non-targeted approaches for the introduction of mutations [95,96,97]. Sequence diversity is introduced randomly into the whole antibody sequence. This also leads to the introduction of deleterious mutations in conserved framework regions and necessitates the selection of a large repertoire to identify improved functional candidates. Error prone PCR is often used in combination with ribosomal display for two reasons: first, a PCR amplification step is already part of the technology and second, large libraries (up to 10^12^) are easy to construct due to the lack of a transformation step. Examples for the use of error prone PCR are numerous: Hanes reported the improvement of the affinity of a hemagglutinin specific scFv by the use of error prone PCR and ribosomal display [9]. Hawkins and coworkers used error prone PCR in combination with phage display resulting in a moderate 4.5-fold increase in affinity of a hapten specific antibody fragment [98]. 

Low and coworkers used the *E. coli* mutator strain mutD5 in combination with subsequent phage display to increase the affinity of a phOx antibody fragment by a factor of 100-fold. While the introduced mutations scatter over the entire antibody sequence, those which confer increased affinity cluster in the CDR regions [99,100,101].

Chain shuffling is another method used for non-targeted diversification. In this approach one of the two antibody chains is replaced by a repertoire while the other chain is kept constant. Marks and coworkers used the method of chain shuffling with a scFv specific for the hapten phOx, selected from a naïve library [52]. The VH of this scFv was combined with a naïve repertoire of V_kappa and V_lambda light chains and the newly selected VL was then combined with a repertoire of the first two CDRs of the VH, leaving the CDR-H3 unaltered. 20-fold affinity improvement by light chain shuffling and further 15-fold by heavy chain shuffling could be achieved. Since then a lot of groups successfully used antibody chain shuffling for affinity improvement resulting e.g., in a 5–6 fold affinity improvement of an erbB2 specific antibody fragment and a 30-fold affinity improvement of a VEGF specific antibody fragment [102,103]. 

A further development is DNA shuffling by random fragmentation and reassembly by PCR, also called sexual PCR [104]. This method involves the digestion and thereby fragmentation of a gene pool of closely related sequences and the subsequent reassembly by PCR leading to shuffling of DNA fragments. This approach can be combined with error prone PCR or mutator *E. coli* strains. Boder and coworkers used this combination of methods together with yeast display to increase the affinity of a scFv specific for fluorescein. A 1,000-fold decreased dissociation rate and a sub-picomolar affinity was observed after maturation [15]. Different combinations of methods lead to good results, e.g., the combination of error prone PCR with chain shuffling resulting in a 22-fold improved affinity of a Fas-specific scFv [99].

### 5.2. Targeted Diversification

Targeted strategies introduce diversity at defined positions that are predicted to contribute to the antigen binding, mainly in the CDR regions, followed by stringent selections. CDR-targeted mutagenesis is advantageous since optimization of these regions is most likely to improve affinity and least likely to create problems with protein stability. In this maturation concept, CDRs are targeted either in a parallel or a sequential fashion. The approaches differ by the localization and amount of diversity introduced. Both of these aspects can already be implemented in the design of the initial library.

The term CDR walking has been used to describe an approach where only limited diversity is introduced in short (4-6) amino acid stretches of a single CDR. Both parallel and sequential CDR walking using degenerated oligonucleotides was used to improve the affinity of a gp120 (HIV antigen) specific antibody [105,106]. For the sequential approach CDR-H1, was randomized first, followed by CDR-H3. For the parallel approach five independent libraries were generated randomizing a single CDR each, followed by combining improved CDRs from antibodies with high affinities after selection. The best affinity improvement was reached using the parallel approach which led to a 420-fold increased affinity [106]. 

One advantage of synthetic libraries is that the requirements for easy diversification can already be implemented in the library e.g. with restriction sites flanking the CDRs. The HuCAL^®^ libraries have restriction site flanking all six CDRs. All CDRs of a given antibody are therefore available for rapid diversification through the use of pre-made maturation cassettes that replace the original CDR. 

The CDR cassettes used for maturation are similar to the design of the initial library: diversity replicating the natural distribution is reproduced faithfully by the TRIM technology to maximize functional library size [85]. Maturations are performed either with a defined, characterized antibody or included into the initial selection using a pool of preselected antibody candidates in a process called RapMAT^®^, which stands for rapid maturation [107,108]. Maturation of a single Fab by diversifying CDR-L3 and CDR-H2 in parallel and combining optimized CDRs led to a 5,000-fold improved affinity of a GM-CSF specific Fab fragment [108]. 

## 6. Conclusions

Over the past 15 years the generation of therapeutic antibodies from combinatorial antibody libraries has been established as a valid alternative to conventional immunization. To isolate candidates from such libraries the antibodies or antibody fragments are displayed on the surface of cells, phages, ribosomes or within the cell. Among those display methods, phage display has the longest history and it is among the technologies most commonly used today. While the choice of the antibody format can be limited by the display method, e.g., to the scFv or the Fab in the case of phage display, library design is in principle independent, as long as sufficient expression properties in the respective host system are ensured. 

Combinatorial antibody libraries differ in design, origin of sequence diversity and method of generation. All three aspects have an impact on the most essential features of a library: its size and functional diversity reflected in the ability to deliver high affinity antibodies with good biophysical properties. Antibody libraries can be classified into three major groups: naïve, semi-synthetic and synthetic libraries. Most libraries aim at maximizing library diversity, but differ in their approaches of maximizing functional size and subsequent developability of the selected molecules. Limitation to a single stable framework or to a selected number of consensus frameworks to ensure good biophysical properties is one trend in this context. Currently there is a trend to be as close to human germline as possible, both in the choice of framework and CDR composition, to avoid potential issues of immunogenicity. 

With the synthetic library of moderate size described by Pini and coworkers, scFvs were selected with monovalent affinities down to 10 nM [57]. Affinities achieved with large semi-synthetic libraries described by Hoet and Söderlind reached the sub-nanomolar range, whereas antibodies in the lower picomolar range have been isolated directly from the fully synthetic HuCAL PLATINUM^®^ library as well as the very large naïve CAT2.0 library. Affinity improvement factors vary widely within each method and are highly dependent on both the antigen as well as the starting molecule. The approaches targeting CDR regions have advantages concerning functional size of maturation libraries and integrity of antibody framework. 

This concept of targeted CDR mutagenesis has been implemented in the design of several synthetic libraries, such as the HuCAL^®^ or Dyax libraries. On the one hand, these findings thus confirm the expected correlation between library size and average affinities. On the other hand, they show that there is more than one good solution for designing an antibody library. Concerning the aspect of affinity, very different library designs are able to deliver comparable results. 

## References

[1] Perelson A.S., Oster G.F. (1979). Theoretical studies of clonal selection: Minimal antibody repertoire size and reliability of self-non-self discrimination. J. Theor. Biol..

[2] Nelson A.L., Dhimolea E., Reichert J.M. (2010). Development trends for human monoclonal antibody therapeutics. Nat. Rev. Drug Discov..

[3] Brooks S.A. (2009). Strategies for analysis of the glycosylation of proteins: Current status and future perspectives. Mol. Biotechnol..

[4] Getts D.R., Getts M.T., McCarthy D.P., Chastain E.M., Miller S.D. (2010). Have we overestimated the benefit of human(ized) antibodies?. MAbs.

[5] Visintin M., Tse E., Axelson H., Rabbitts T.H., Cattaneo A. (1999). Selection of antibodies for intracellular function using a two-hybrid *in vivo* system. Proc. Natl. Acad. Sci USA.

[6] Auf der Maur A., Tissot K., Barberis A. (2004). Antigen-independent selection of intracellular stable antibody frameworks. Methods.

[7] Mossner E., Koch H., Plückthun A. (2001). Fast selection of antibodies without antigen purification: adaptation of the protein fragment complementation assay to select antigen-antibody pairs. J. Mol. Biol..

[8] Mattheakis L.C., Bhatt R.R., Dower W.J. (1994). An *in vitro* polysome display system for identifying ligands from very large peptide libraries. Proc. Natl. Acad. Sci. USA.

[9] Hanes J., Plückthun A. (1997). *In vitro* selection and evolution of functional proteins by using ribosome display. Proc. Natl. Acad. Sci. USA.

[10] Boder E.T., Wittrup K.D. (1997). Yeast surface display for screening combinatorial polypeptide libraries. Nat. Biotechnol..

[11] Feldhaus M.J., Siegel R.W., Opresko L.K., Coleman J.R., Feldhaus J.M., Yeung Y.A., Cochran J.R., Heinzelman P., Colby D., Swers J. (2003). Flow-cytometric isolation of human antibodies from a nonimmune Saccharomyces cerevisiae surface display library. Nat. Biotechnol..

[12] Weaver-Feldhaus J.M., Lou J., Coleman J.R., Siegel R.W., Marks J.D., Feldhaus M.J. (2004). Yeast mating for combinatorial Fab library generation and surface display. FEBS Lett..

[13] Feldhaus M.J. Human Antibody Discovery and Optimization in Yeast. Presented at PEGS Protein Engineering Summit, 17-21 May 2010.

[14] Beerli R.R., Bauer M., Buser R.B., Gwerder M., Muntwiler S., Maurer P., Saudan P., Bachmann M.F. (2008). Isolation of human monoclonal antibodies by mammalian cell display. Proc. Natl. Acad. Sci. USA.

[15] Boder E.T., Midelfort K.S., Wittrup K.D. (2000). Directed evolution of antibody fragments with monovalent femtomolar antigen-binding affinity. Proc. Natl. Acad. Sci. USA.

[16] Smith G.P. (1985). Filamentous fusion phage: Novel expression vectors that display cloned antigens on the virion surface. Science.

[17] Clackson T., Wells J.A. (1994). *In vitro* selection from protein and peptide libraries. Trends Biotechnol..

[18] Griffiths A.D., Malmqvist M., Marks J.D., Bye J.M., Embleton M.J., McCafferty J., Baier M., Holliger K.P., Gorick B.D., Hughes-Jones N.C. (1993). Human anti-self antibodies with high specificity from phage display libraries. EMBO J..

[19] Duenas M., Borrebaeck C.A. (1995). Novel helper phage design: intergenic region affects the assembly of bacteriophages and the size of antibody libraries. FEMS Microbiol. Lett..

[20] Rakonjac J., Jovanovic G., Model P. (1997). Filamentous phage infection-mediated gene expression: Construction and propagation of the gIII deletion mutant helper phage R408d3. Gene.

[21] Rondot S., Koch J., Breitling F., Dübel S. (2001). A helper phage to improve single-chain antibody presentation in phage display. Nat. Biotechnol..

[22] Kramer R.A., Cox F., van der Horst M., van der Oudenrijn S., Res P.C., Bia J., Logtenberg T., de Kruif J. (2003). A novel helper phage that improves phage display selection efficiency by preventing the amplification of phages without recombinant protein. Nucleic Acids Res..

[23] Soltes G., Barker H., Marmai K., Pun E., Yuen A., Wiersma E.J. (2003). A new helper phage and phagemid vector system improves viral display of antibody Fab fragments and avoids propagation of insert-less virions. J. Immunol. Methods.

[24] Baek H., Suk K.H., Kim Y.H., Cha S. (2002). An improved helper phage system for efficient isolation of specific antibody molecules in phage display. Nucleic Acids Res..

[25] Burton D.R., Barbas C.F., Persson M.A., Koenig S., Chanock R.M., Lerner R.A. (1991). A large array of human monoclonal antibodies to type 1 human immunodeficiency virus from combinatorial libraries of asymptomatic seropositive individuals. Proc. Natl. Acad. Sci. USA.

[26] Marks J.D., Hoogenboom H.R., Bonnert T.P., McCafferty J., Griffiths A.D., Winter G. (1991). By-passing immunization. Human antibodies from V-gene libraries displayed on phage. J. Mol. Biol..

[27] Vaughan T.J., Williams A.J., Pritchard K., Osbourn J.K., Pope A.R., Earnshaw J.C., McCafferty J., Hodits R.A., Wilton J., Johnson K.S. (1996). Human antibodies with sub-nanomolar affinities isolated from a large non-immunized phage display library. Nat. Biotechnol..

[28] Bradbury A.R., Marks J.D. (2004). Antibodies from phage antibody libraries. J. Immunol. Methods.

[29] Jung S., Honegger A., Plückthun A. (1999). Selection for improved protein stability by phage display. J. Mol. Biol..

[30] Persson M.A., Caothien R.H., Burton D.R. (1991). Generation of diverse high-affinity human monoclonal antibodies by repertoire cloning. Proc. Natl. Acad. Sci. USA.

[31] Röthlisberger D., Honegger A., Plückthun A. (2005). Domain interactions in the Fab fragment: A comparative evaluation of the single-chain Fv and Fab format engineered with variable domains of different stability. J. Mol. Biol..

[32] Ward E.S., Gussow D., Griffiths A.D., Jones P.T., Winter G. (1989). Binding activities of a repertoire of single immunoglobulin variable domains secreted from Escherichia coli. Nature.

[33] Ewert S., Cambillau C., Conrath K., Plückthun A. (2002). Biophysical properties of camelid V(HH) domains compared to those of human V(H)3 domains. Biochemistry.

[34] Dumoulin M., Conrath K., Van M.A., Meersman F., Heremans K., Frenken L.G., Muyldermans S., Wyns L., Matagne A. (2002). Single-domain antibody fragments with high conformational stability. Protein Sci..

[35] Davies J., Riechmann L. (1996). Affinity improvement of single antibody VH domains: Residues in all three hypervariable regions affect antigen binding. Immunotechnology..

[36] Davies J., Riechmann L. (1996). Single antibody domains as small recognition units: Design and *in vitro* antigen selection of camelized, human VH domains with improved protein stability. Protein Eng..

[37] Davies J., Riechmann L. (1995). Antibody VH domains as small recognition units. Biotechnology (N.Y.).

[38] Davies J., Riechmann L. (1994). Camelising' human antibody fragments: NMR studies on VH domains. FEBS Lett..

[39] Riechmann L. (1996). Rearrangement of the former VL interface in the solution structure of a camelised, single antibody VH domain. J. Mol. Biol..

[40] Riechmann L., Muyldermans S. (1999). Single domain antibodies: comparison of camel VH and camelised human VH domains. J. Immunol. Methods.

[41] Muyldermans S., Cambillau C., Wyns L. (2001). Recognition of antigens by single-domain antibody fragments: the superfluous luxury of paired domains. Trends Biochem. Sci..

[42] Muyldermans S. (2001). Single domain camel antibodies: current status. J. Biotechnol..

[43] Desmyter A., Transue T.R., Ghahroudi M.A., Thi M.H., Poortmans F., Hamers R., Muyldermans S., Wyns L. (1996). Crystal structure of a camel single-domain VH antibody fragment in complex with lysozyme. Nat. Struct. Biol..

[44] Desmyter A., Decanniere K., Muyldermans S., Wyns L. (2001). Antigen specificity and high affinity binding provided by one single loop of a camel single-domain antibody. J. Biol. Chem..

[45] Desmyter A., Spinelli S., Payan F., Lauwereys M., Wyns L., Muyldermans S., Cambillau C. (2002). Three camelid VHH domains in complex with porcine pancreatic alpha-amylase. Inhibition and versatility of binding topology. J. Biol. Chem..

[46] Lauwereys M., Arbabi G.M., Desmyter A., Kinne J., Holzer W., De G.E., Wyns L., Muyldermans S. (1998). Potent enzyme inhibitors derived from dromedary heavy-chain antibodies. EMBO J..

[47] De Genst E., Silence K., Decanniere K., Conrath K., Loris R., Kinne J., Muyldermans S., Wyns L. (2006). Molecular basis for the preferential cleft recognition by dromedary heavy-chain antibodies. Proc. Natl. Acad. Sci. USA.

[48] Chapman A.P., Antoniw P., Spitali M., West S., Stephens S., King D.J. (1999). Therapeutic antibody fragments with prolonged *in vivo* half-lives. Nat. Biotechnol..

[49] Lee L.S., Conover C., Shi C., Whitlow M., Filpula D. (1999). Prolonged circulating lives of single-chain Fv proteins conjugated with polyethylene glycol: a comparison of conjugation chemistries and compounds. Bioconjug.Chem..

[50] Huse W.D., Sastry L., Iverson S.A., Kang A.S., Alting-Mees M., Burton D.R., Benkovic S.J., Lerner R.A. (1989). Generation of a large combinatorial library of the immunoglobulin repertoire in phage lambda. Science.

[51] Kramer R.A., Marissen W.E., Goudsmit J., Visser T.J., Clijsters-Van der Horst M., Bakker A.Q., de Jong M., Jongeneelen M., Thijsse S., Backus H.H. (2005). The human antibody repertoire specific for rabies virus glycoprotein as selected from immune libraries. Eur. J. Immunol..

[52] Marks J.D., Griffiths A.D., Malmqvist M., Clackson T.P., Bye J.M., Winter G. (1992). By-passing immunization: building high affinity human antibodies by chain shuffling. Biotechnology (N.Y.).

[53] Lloyd C., Lowe D., Edwards B., Welsh F., Dilks T., Hardman C., Vaughan T. (2009). Modelling the human immune response: performance of a 1011 human antibody repertoire against a broad panel of therapeutically relevant antigens. Protein Eng. Des. Sel..

[54] Hoogenboom H.R. (2002). Overview of antibody phage-display technology and its applications. Methods Mol. Biol..

[55] Nissim A., Hoogenboom H.R., Tomlinson I.M., Flynn G., Midgley C., Lane D., Winter G. (1994). Antibody fragments from a 'single pot' phage display library as immunochemical reagents. EMBO J..

[56] de Kruif J., Boel E., Logtenberg T. (1995). Selection and application of human single chain Fv antibody fragments from a semi-synthetic phage antibody display library with designed CDR3 regions. J. Mol. Biol..

[57] Pini A., Viti F., Santucci A., Carnemolla B., Zardi L., Neri P., Neri D. (1998). Design and use of a phage display library. Human antibodies with subnanomolar affinity against a marker of angiogenesis eluted from a two-dimensional gel. J. Biol. Chem..

[58] Carlsson R., Söderlind E. (2001). n-CoDeR concept: unique types of antibodies for diagnostic use and therapy. Expert Rev. Mol. Diagn..

[59] Söderlind E., Strandberg L., Jirholt P., Kobayashi N., Alexeiva V., Aberg A.M., Nilsson A., Jansson B., Ohlin M., Wingren C., Danielsson L., Carlsson R., Borrebaeck C.A. (2000). Recombining germline-derived CDR sequences for creating diverse single-framework antibody libraries. Nat. Biotechnol..

[60] Lee C.V., Liang W.C., Dennis M.S., Eigenbrot C., Sidhu S.S., Fuh G. (2004). High-affinity human antibodies from phage-displayed synthetic Fab libraries with a single framework scaffold. J. Mol. Biol..

[61] Liang W.C., Dennis M.S., Stawicki S., Chanthery Y., Pan Q., Chen Y., Eigenbrot C., Yin J., Koch A.W., Wu X., Ferrara N., Bagri A., Tessier-Lavigne M., Watts R.J., Wu Y. (2007). Function blocking antibodies to neuropilin-1 generated from a designed human synthetic antibody phage library. J. Mol. Biol..

[62] Griffiths A.D., Williams S.C., Hartley O., Tomlinson I.M., Waterhouse P., Crosby W.L., Kontermann R.E., Jones P.T., Low N.M., Allison T.J. (1994). Isolation of high affinity human antibodies directly from large synthetic repertoires. EMBO J..

[63] Hoet R.M., Cohen E.H., Kent R.B., Rookey K., Schoonbroodt S., Hogan S., Rem L., Frans N., Daukandt M., Pieters H. (2005). Generation of high-affinity human antibodies by combining donor-derived and synthetic complementarity-determining-region diversity. Nat. Biotechnol..

[64] Rothe C., Urlinger S., Lohning C., Prassler J., Stark Y., Jager U., Hubner B., Bardroff M., Pradel I., Boss M. (2008). The human combinatorial antibody library HuCAL GOLD combines diversification of all six CDRs according to the natural immune system with a novel display method for efficient selection of high-affinity antibodies. J. Mol. Biol..

[65] Waterhouse P., Griffiths A.D., Johnson K.S., Winter G. (1993). Combinatorial infection and *in vivo* recombination: a strategy for making large phage antibody repertoires. Nucleic Acids Res..

[66] Sblattero D., Bradbury A. (2000). Exploiting recombination in single bacteria to make large phage antibody libraries. Nat. Biotechnol..

[67] Seehaus T., Breitling F., Dübel S., Klewinghaus I., Little M. (1992). A vector for the removal of deletion mutants from antibody libraries. Gene.

[68] Azzazy H.M., Highsmith W.E. (2002). Phage display technology: clinical applications and recent innovations. Clin. Biochem..

[69] Benhar I. (2007). Design of synthetic antibody libraries. Expert Opin. Biol. Ther..

[70] de Carvalho N.C., Williamson R.A., Parren P.W., Lundkvist A., Burton D.R., Bjorling E. (2002). Neutralizing human Fab fragments against measles virus recovered by phage display. J. Virol..

[71] Zebedee S.L., Barbas C.F., Hom Y.L., Caothien R.H., Graff R., DeGraw J., Pyati J., LaPolla R., Burton D.R., Lerner R.A. (1992). Human combinatorial antibody libraries to hepatitis B surface antigen. Proc. Natl. Acad. Sci. USA.

[72] Cai X., Garen A. (1995). Anti-melanoma antibodies from melanoma patients immunized with genetically modified autologous tumor cells: selection of specific antibodies from single-chain Fv fusion phage libraries. Proc. Natl. Acad. Sci. USA.

[73] Hamers-Casterman C., Atarhouch T., Muyldermans S., Robinson G., Hamers C., Songa E.B., Bendahman N., Hamers R. (1993). Naturally occurring antibodies devoid of light chains. Nature.

[74] Muyldermans S., Atarhouch T., Saldanha J., Barbosa J.A., Hamers R. (1994). Sequence and structure of VH domain from naturally occurring camel heavy chain immunoglobulins lacking light chains. Protein Eng..

[75] Muyldermans S., Lauwereys M. (1999). Unique single-domain antigen binding fragments derived from naturally occurring camel heavy-chain antibodies. J. Mol. Recognit..

[76] Vincke C., Loris R., Saerens D., Martinez-Rodriguez S., Muyldermans S., Conrath K. (2009). General strategy to humanize a camelid single-domain antibody and identification of a universal humanized nanobody scaffold. J. Biol. Chem..

[77] Sheets M.D., Amersdorfer P., Finnern R., Sargent P., Lindquist E., Schier R., Hemingsen G., Wong C., Gerhart J.C., Marks J.D. (1998). Efficient construction of a large nonimmune phage antibody library: the production of high-affinity human single-chain antibodies to protein antigens. Proc. Natl. Acad. Sci. USA.

[78] De Haard H.J. (2002). Construction of large naive Fab libraries. Methods Mol. Biol..

[79] Hoogenboom H.R., Winter G. (1992). By-passing immunisation. Human antibodies from synthetic repertoires of germline VH gene segments rearranged *in vitro*. J. Mol. Biol..

[80] Schoonbroodt S., Steukers M., Viswanathan M., Frans N., Timmermans M., Wehnert A., Nguyen M., Ladner R.C., Hoet R.M. (2008). Engineering antibody heavy chain CDR3 to create a phage display Fab library rich in antibodies that bind charged carbohydrates. J. Immunol..

[81] Knappik A., Ge L., Honegger A., Pack P., Fischer M., Wellnhofer G., Hoess A., Wolle J., Plückthun A., Virnekas B. (2000). Fully synthetic human combinatorial antibody libraries (HuCAL) based on modular consensus frameworks and CDRs randomized with trinucleotides. J. Mol. Biol..

[82] Rauchenberger R., Borges E., Thomassen-Wolf E., Rom E., Adar R., Yaniv Y., Malka M., Chumakov I., Kotzer S., Resnitzky D., Knappik A., Reiffert S., Prassler J., Jury K., Waldherr D., Bauer S., Kretzschmar T., Yayon A., Rothe C. (2003). Human combinatorial Fab library yielding specific and functional antibodies against the human fibroblast growth factor receptor 3. J. Biol. Chem..

[83] Steipe B., Schiller B., Plückthun A., Steinbacher S. (1994). Sequence statistics reliably predict stabilizing mutations in a protein domain. J. Mol. Biol..

[84] Krebs B., Rauchenberger R., Reiffert S., Rothe C., Tesar M., Thomassen E., Cao M., Dreier T., Fischer D., Hoss A., Inge L., Knappik A., Marget M., Pack P., Meng X.Q., Schier R., Sohlemann P., Winter J., Wolle J., Kretzschmar T. (2001). High-throughput generation and engineering of recombinant human antibodies. J. Immunol. Methods.

[85] Virnekas B., Ge L., Plückthun A., Schneider K.C., Wellnhofer G., Moroney S.E. (1994). Trinucleotide phosphoramidites: ideal reagents for the synthesis of mixed oligonucleotides for random mutagenesis. Nucleic Acids Res..

[86] Sidhu S.S., Li B., Chen Y., Fellouse F.A., Eigenbrot C., Fuh G. (2004). Phage-displayed antibody libraries of synthetic heavy chain complementarity determining regions. J. Mol. Biol..

[87] Fellouse F.A., Wiesmann C., Sidhu S.S. (2004). Synthetic antibodies from a four-amino-acid code: a dominant role for tyrosine in antigen recognition. Proc. Natl. Acad. Sci. USA.

[88] Fellouse F.A., Li B., Compaan D.M., Peden A.A., Hymowitz S.G., Sidhu S.S. (2005). Molecular recognition by a binary code. J. Mol. Biol..

[89] Fellouse F.A., Barthelemy P.A., Kelley R.F., Sidhu S.S. (2006). Tyrosine plays a dominant functional role in the paratope of a synthetic antibody derived from a four amino acid code. J. Mol. Biol..

[90] Tanha J., Xu P., Chen Z., Ni F., Kaplan H., Narang S.A., MacKenzie C.R. (2001). Optimal design features of camelized human single-domain antibody libraries. J. Biol. Chem..

[91] Reiter Y., Schuck P., Boyd L.F., Plaksin D. (1999). An antibody single-domain phage display library of a native heavy chain variable region: isolation of functional single-domain VH molecules with a unique interface. J. Mol. Biol..

[92] Jespers L., Schon O., James L.C., Veprintsev D., Winter G. (2004). Crystal structure of HEL4, a soluble, refoldable human V(H) single domain with a germ-line scaffold. J. Mol. Biol..

[93] Jespers L., Schon O., Famm K., Winter G. (2004). Aggregation-resistant domain antibodies selected on phage by heat denaturation. Nat. Biotechnol..

[94] Thie H., Voedisch B., Dübel S., Hust M., Schirrmann T. (2009). Affinity maturation by phage display. Methods Mol. Biol..

[95] Cadwell R.C., Joyce G.F. (1994). Mutagenic PCR. PCR Methods Appl..

[96] Coia G., Hudson P.J., Irving R.A. (2001). Protein affinity maturation *in vivo* using E. coli mutator cells. J. Immunol. Methods.

[97] Low N.M., Holliger P.H., Winter G. (1996). Mimicking somatic hypermutation: affinity maturation of antibodies displayed on bacteriophage using a bacterial mutator strain. J. Mol. Biol..

[98] Hawkins R.E., Russell S.J., Winter G. (1992). Selection of phage antibodies by binding affinity. Mimicking affinity maturation. J. Mol. Biol..

[99] Chodorge M., Fourage L., Ravot G., Jermutus L., Minter R. (2008). *In vitro* DNA recombination by L-Shuffling during ribosome display affinity maturation of an anti-Fas antibody increases the population of improved variants. Protein Eng. Des. Sel..

[100] Dufner P., Jermutus L., Minter R.R. (2006). Harnessing phage and ribosome display for antibody optimisation. Trends Biotechnol..

[101] Schwesinger F., Ros R., Strunz T., Anselmetti D., Guntherodt H.J., Honegger A., Jermutus L., Tiefenauer L., Plückthun A. (2000). Unbinding forces of single antibody-antigen complexes correlate with their thermal dissociation rates. Proc. Natl. Acad. Sci. USA.

[102] Lu D., Shen J., Vil M.D., Zhang H., Jimenez X., Bohlen P., Witte L., Zhu Z. (2003). Tailoring *in vitro* selection for a picomolar affinity human antibody directed against vascular endothelial growth factor receptor 2 for enhanced neutralizing activity. J. Biol. Chem..

[103] Schier R., Bye J., Apell G., McCall A., Adams G.P., Malmqvist M., Weiner L.M., Marks J.D. (1996). Isolation of high-affinity monomeric human anti-c-erbB-2 single chain Fv using affinity-driven selection. J. Mol. Biol..

[104] Stemmer W.P. (1994). DNA shuffling by random fragmentation and reassembly: *in vitro* recombination for molecular evolution. Proc. Natl. Acad. Sci. USA.

[105] Barbas C.F., Hu D., Dunlop N., Sawyer L., Cababa D., Hendry R.M., Nara P.L., Burton D.R. (1994). *In vitro* evolution of a neutralizing human antibody to human immunodeficiency virus type 1 to enhance affinity and broaden strain cross-reactivity. Proc. Natl. Acad. Sci. USA.

[106] Yang W.P., Green K., Pinz-Sweeney S., Briones A.T., Burton D.R., Barbas C.F. (1995). CDR walking mutagenesis for the affinity maturation of a potent human anti-HIV-1 antibody into the picomolar range. J. Mol. Biol..

[107] Prassler J., Steidl S., Urlinger S. (2009). *In vitro* affinity maturation of HuCAL antibodies: complementarity determining region exchange and RapMAT technology. Immunotherapy.

[108] Steidl S., Ratsch O., Brocks B., Durr M., Thomassen-Wolf E. (2008). *In vitro* affinity maturation of human GM-CSF antibodies by targeted CDR-diversification. Mol. Immunol..

